# Multi-Geometric Reasoning Network for Insulator Defect Detection of Electric Transmission Lines

**DOI:** 10.3390/s22166102

**Published:** 2022-08-15

**Authors:** Yongjie Zhai, Zhedong Hu, Qianming Wang, Qiang Yang, Ke Yang

**Affiliations:** 1 Automation Department, North China Electric Power University, Baoding 071003, China; 2 College of Electrical Engineering, Zhejiang University, Hangzhou 310027, China

**Keywords:** multi-geometric reasoning, insulator defect detection, deep learning, graph convolutional neural network

## Abstract

To address the challenges in the unmanned system-based intelligent inspection of electric transmission line insulators, this paper proposed a multi-geometric reasoning network (MGRN) to accurately detect insulator geometric defects based on aerial images with complex backgrounds and different scales. The spatial geometric reasoning sub-module (SGR) was developed to represent the spatial location relationship of defects. The appearance geometric reasoning sub-module (AGR) and the parallel feature transformation (PFT) sub-module were adopted to obtain the appearance geometric features from the real samples. These multi-geometric features can be fused with the original visual features to identify and locate the insulator defects. The proposed solution is assessed through experiments against the existing solutions and the numerical results indicate that it can significantly improve the detection accuracy of multiple insulator defects using the aerial images.

## 1. Introduction

**Figure 1:**
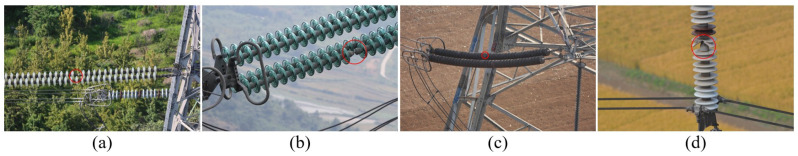
Aerial insulator images with geometric defects (red circle). (**a**,**b**): missing defects; (**c**,**d**): damaged defects.

The electric power transmission line insulators are widely used in power systems to fix and insulate electrical equipment [1]. Due to the long-term exposure to the natural environment, the insulators are susceptible to damage, and the chance of failure of damaged insulators will increase, which will directly threaten the stability and safety of transmission lines [2,3]. Therefore, timely detection of insulator defects is crucial. Common insulator defects include damaged [4] and missing [5]. The geometric defect of an object is defined as the change of its geometry caused by natural or human factors, as shown in Figure 1.

A common approach for insulator defect detection under these two categories is to capture aerial images of insulators by unmanned aerial vehicles (UAVs), which are then analyzed and processed using computer vision techniques [6,7,8]. Several recent studies have demonstrated the effectiveness of machine learning techniques in insulator detection. For instance, the authors in [9] achieved the detection of damaged insulator strings by extracting the shape and texture information of the insulator and improving the watershed algorithm. The study in [10] assessed the damage level of insulators by extracting insulator features with wavelet transform and then analyzing the insulator condition through the support vector machine. The authors in [11] represented the local features by introducing multi-scale and multi-feature descriptors, and then proposed a coarse-to-fine matching strategy to achieve insulator detection based on the spatial order features in the local features. Although these studies have achieved certain considerable results, machine learning approaches for vision processing by designing feature extraction modules are difficult to achieve better adaptability and robustness.

In recent years, advances in artificial intelligence enable deep-learning-based techniques to be adopted for insulator defect detection. In [12], the authors proposed an insulator missing detection network with compact feature space based on a stochastic configuration network and feedback transfer learning mechanism, and achieved adaptive adjustment of the depth feature space; The work in [13] introduced a deep neural network with cascade structure, which converted the insulator defect problem into a two-level object detection problem and realized the localization and identification of insulator missing. The authors in [14] improved the YOLOv3 model by employing Spatial Pyramid Pooling network (SPP) and multiscale prediction network and carried out training on a large number of insulator missing samples, achieving insulator defect detection under different aerial photography backgrounds. These insulator defect detections mainly focus on one single defect, i.e., missing transmission line insulators, and have achieved satisfactory results. Such a defect occurs frequently in overhead transmission lines in practice, and hence a large number of sample images are available that can be used for deep learning models.

Unfortunately, the sample images are scarce in the actual detection environment for defects such as damage d, flashover [15], and dirty [16]. Moreover, the morphological features presented by these defects are more diverse and complex than the missing defect, making it difficult for deep learning models to perform better. The work in [17] proposed a multiscale residual neural network that achieved insulator damage detection in a single background through rich spatial correlation and channel correlation. In [18] the authors implemented a state assessment for the existence of ice, snow, and water on the insulator surface based on the YOLOv2 model through data expansion. The authors in [19] realized the detection of insulator damaged defects by improving Regional Proposal Network (RPN) and adding the improved ResNeSt [20] for feature extraction. In general, the existing research is mainly from the perspective of data augmentation of defect samples, by increasing the sample quantity and then completing the training for the deep learning model to realize the detection of scarcity defects. However, the application of deep learning technology based on a small number of samples to defect detection of transmission line insulators is still at the exploratory stage. Furthermore, much research effort has been made in the field of machine learning-based detection methods. The current object detection frameworks are mainly divided into one-stage detectors and two-stage detectors. The one-stage detectors are mainly based on SSD [21] and YOLO series [22], and the representative frameworks presented by the two-stage detectors include Fast R-CNN [23], Faster R-CNN [24], and Mask R-CNN [25]. However, when used in practical industrial scenarios, even high-performance object detectors have difficulty showing great performance. For example, detectors with excellent performance on public datasets often are difficult to work reliably in power systems [26] or transportation systems [27] with complex scenarios.

However, there still remains a set of technical challenges in deep learning for defect detection of insulator s. The three challenges are as follows: (1) Complex background. As a basic electrical insulation device, insulators are widely applied in fields, woods, and buildings. (2) Various types. The existing insulators mainly include glass insulators, ceramic insulators, and composite insulators, which make the defects under different insulators more diverse. (3) Different scales. Insulator defects show multi-scale in appearance. For example, the scale of missing is relatively large, while the scale of damage is small. In order to better accomplish the detection of geometric defects of insulators. We propose a Multi-Geometric Reasoning Networks (MGRN), which fully taps into the geometric information of defect samples and the spatial location information of defects to address the three challenges in insulator defect detection. The detection accuracy of insulator geometric defects is significantly improved. The main technical contributions of this work can be summarized as follows:

1. Aiming at the different challenges in the defect detection of insulators, we construct two different types of geometric features and propose a multi-geometric reasoning network model (MGRN) to integrate them. This model can effectively improve the detection accuracy of insulator defects on transmission lines, and the recognition effect is remarkable, especially for some hard-detection geometric defects.
2. The appearance geometric reasoning (AGR) module is used to extract artificial defect sample features. The parallel feature transformation (PFT) module can enable the feature to be used in the real defect samples and extract the appearance geometric feature of the real defect samples. The spatial geometric reasoning (SGR) module is used to extract spatial geometric features of real defect samples. Thus, the multi-geometric features can be better integrated into the deep learning model.
3. The model can achieve better performance on a small number of samples, as well as a better improvement in insulator damage defect detection. It also provides a new idea for multi-scale object detection with few samples.

The rest of the paper is organized as follows. Section 2 describes the appearance geometric reasoning model, and the parallel feature transformation, the proposed spatial geometric reasoning model. Section 3 assesses the performance of the solution and presents the numerical results. Finally, the conclusive remarks are given in Section 4.

## 2. Proposed MGRN Based Solution

### 2.1. System Overview

**Figure 2:**
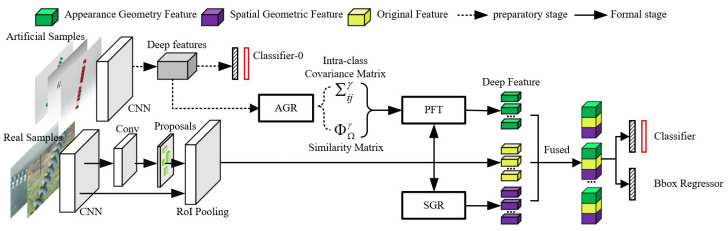
Architecture of the proposed MGRN. In the preparatory phase, the appearance geometric features of the artificial samples are modelled as intra-class covariance matrices and similarity matrices by the AGR sub-module. In the formal phase, the original features are extracted from the real samples (using CNN and RoI Pooling); the appearance geometric and spatial geometric features are extracted from the real samples (using SGR sub-module and PFT sub-module); finally the obtained features are fused from the real samples.

In this article, insulator geometry defect detection is regarded as an instance-level task. The proposed MGRN is built on the Faster R-CNN [24] as the detector framework. The architecture of the proposed approach includes three major components: (1) appearance geometric reasoning sub-module (AGR) for artificial sample defect feature information extraction; (2) parallel feature transformation sub-module (PFT) for mapping artificial sample features to real sample feature space; (3) spatial geometric reasoning sub-module (SGR) for real sample space information extraction, as illustrated in Figure 2.

The MGRN network involves a preparatory phase and a formal phase. In the preparatory phase, the appearance geometric features of the artificial samples are modelled as intra-class covariance matrices and similarity matrices by the AGR sub-module. In the formal phase, our algorithm produces feature map and region proposals from input real images based on the CNN backbone and region proposal network (RPN). Next, the PFT and SGR sub-modules are utilized to respectively generate appearance geometric features and spatial geometric features. Finally, these feature maps are fused. The new method MGRN effectively introduces the geometric information from the space and exterior points of view and improves the accuracy of insulator defects detection.

### 2.2. Appearance Geometric Reasoning

**Figure 3:**
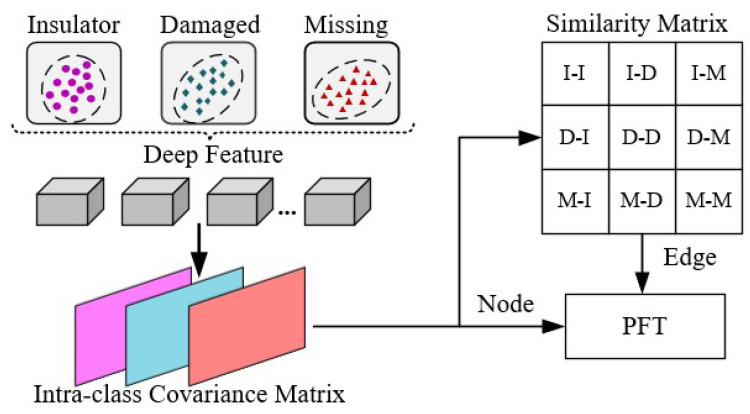
Architecture of the AGR model.

**Figure 4:**
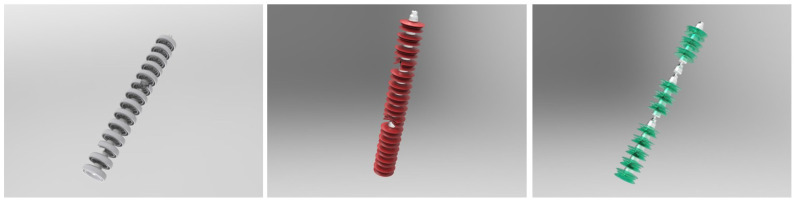
Artificial defect sample images of insulators (glass, ceramic and composite insulators).

In aerial images of insulator defects, due to the scarcity of defect sample data, it is difficult for data-driven deep learning models to achieve good results. Therefore, an appearance geometric reasoning sub-module is proposed which uses artificial defect samples to assist in extracting defect appearance features, as shown in Figure 3. A large number of artificial 2D solid-color background images are generated by constructing 3D artificial samples, as shown in Figure 4. These artificial defect images have the advantages of a simple background, and obvious and diverse defect features so that we can better obtain the appearance and geometric features of the defects.

**Figure 5:**
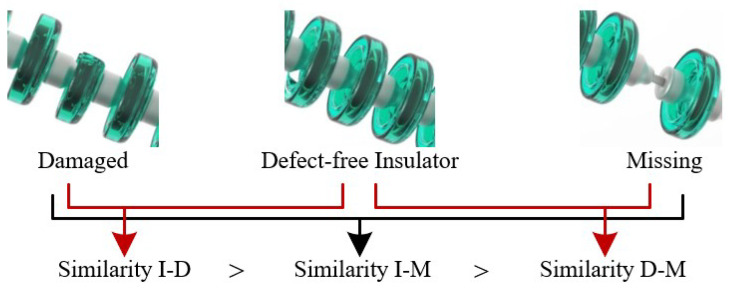
Similarity ranking of insulators with damaged and missing defects compared with the defect-free insulator.

First, the artificial defect images are delivered to a convolutional neural network for extracting deep features $f=F\left(x,\Theta_{F}\right)\in R^{3\times 512\times 512}$, where *x* is the input image; F(•) is the parameterized feature extractor of $\Theta_{F}$. The deep features *f* include the distribution of three main types of features, which are the main body features of the insulator (I), insulator damaged features (D), and insulator missing features (M). It is obvious that the feature similarity of I-D is greater than that of I-M and the feature similarity of I-M is greater than that of D-M in terms of physical geometry ($S_{I-D}>S_{I-M}>S_{D-M}$), as shown in Figure 5. It can be observed that whether the appearance of geometric features of artificial defect samples can be fully extracted depends on the training results of the feature extraction network. For this purpose, we choose the deep features with accuracy γ(95%≤γ≤99%), and γ is a hyperparameter. Next, the intra-class covariance matrices $\Sigma_{ij}^{\gamma }$ of the three types of features are calculated to represent the distribution of the appearance geometric features of the insulator body, insulator damaged, and insulator missing. The calculation formula is as follows:

(1) $$\Sigma_{ij}^{\gamma }=cov\left(f_{i}^{\gamma },f_{j}^{\gamma }\right),\left(i\neq j;i,j\in 1,2,3\right)$$

where $\Sigma_{ij}^{\gamma }=\left\{\Sigma_{I}^{\gamma },\Sigma_{D}^{\gamma },\Sigma_{M}^{\gamma }\left|\Sigma_{ij}^{\gamma }\in R^{1\times 512\times 512}\right.\right\}$.

Then, the cosine similarity $\Omega_{\alpha ,\beta }^{\gamma }$ of the appearance geometric features $\Sigma_{I}^{\gamma }$, $\Sigma_{D}^{\gamma }$ and $\Sigma_{M}^{\gamma }$ are calculated to represent the geometric similarity of the physical appearance of the insulator and geometric defects on the instance-level image. The calculation formula is as follows:

(2) $$\Omega_{\alpha ,\beta }^{\gamma }=\frac{\sum_{\alpha }^{\gamma }\cdot \sum_{\beta }^{\gamma }}{∥\sum_{\alpha }^{\gamma }∥∥\sum_{\beta }^{\gamma }∥},\left(\alpha ,\beta \in \left\{I,D,M\right\}\right)$$

At this point, we get the two parameters delivered to the PFT sub-module of the formal phase for graph reasoning, which are node features $\Sigma_{I}^{\gamma }$, $\Sigma_{D}^{\gamma }$ and $\Sigma_{M}^{\gamma }$ (the appearance geometric features of artificially defect samples) and node relationship matrix $\Phi_{\Omega }^{\gamma }$ (the similarity matrix of appearance geometric features). Finally, we ensure the convergence of the network parameters during the training of artificially defect samples by a cross-entropy loss function. The calculation formula is as follows:

(3) $$L_{1}=-\sum_{i=1}^{n}p\left(x_{i}\right)log\left(q\left(x_{i}\right)\right)$$

where *n* is the number of samples; $p(x_{i})$ is the artificial defect sample label, and $q(x_{i})$ is the probability of the defect category predicted by the model.

In this section of appearance geometric reasoning, we describe how to obtain information about the appearance geometric features and similarity of defect samples. However, the information is obtained based on artificial samples. How to apply the information to the detection of real defect samples is a problem worth considering. To solve the problem, the parallel feature transformation sub-module (PFT) is designed for Artificial-Real transformation.

### 2.3. Parallel Feature Transformation

**Figure 6:**
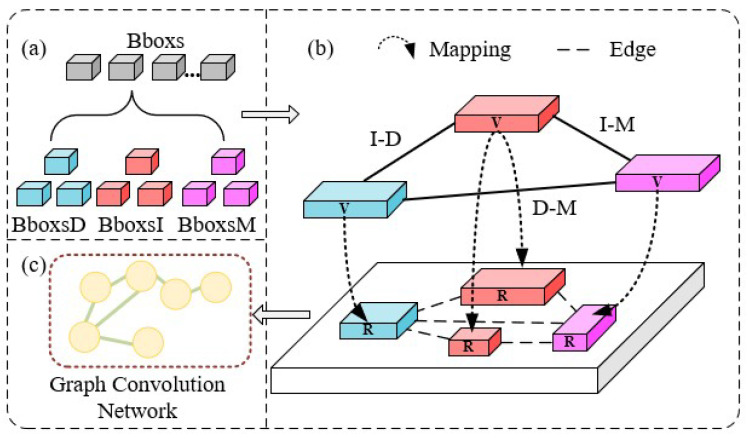
Architecture of the PFT model. (**a**) shows that the box contains category information and feature information; (**b**) shows a mapping mechanism; (**c**) shows the process of using graph neural network for the appearance geometric reasoning of real samples.

In this section, the parallel feature transformation sub-module (PFT) will be described in detail, as shown in Figure 6.

In the formal phase, the object regional proposals (Bboxs) are obtained from the real samples through CNN, RPN, and RoI Pooling in turn. The Bboxs contain the category information and feature information, as shown in Figure 6a. Then, we map the appearance geometric features $\Sigma_{I}^{\gamma }$, $\Sigma_{D}^{\gamma }$ and $\Sigma_{M}^{\gamma }$ and the similarity matrix $\Phi_{\Omega }^{\gamma }$ generated by the AGR sub-module to the object regional proposals with the same category labels by the mapping mechanism *g*. The purpose is to obtain the appearance geometric features $\Sigma_{I}^{R\gamma }$, $\Sigma_{D}^{R\gamma }$ and $\Sigma_{M}^{R\gamma }$ and the similarity matrix $\Phi_{\Omega }^{R\gamma }$ that can be used to assist in the detection of real samples, as shown in Figure 6b. The calculation formula is as follows:

(4) $$g:\hbar \left(\Sigma_{ij}^{\gamma },\Phi_{\Omega }^{\gamma }\right)\to \hbar^{R}\left(\Sigma_{ij}^{R\gamma },\Phi_{\Omega }^{R\gamma }\right)$$

where $g^{-1}\left(\hbar^{R}\right)\subseteq \hbar$; *g* is a unidirectional surjection function; *ℏ* and $\hbar^{R}$ correspond to the artificial sample feature space and the real sample feature space, respectively.

Finally, the appearance geometric features $\Sigma_{I}^{R\gamma }$, $\Sigma_{D}^{R\gamma }$ and $\Sigma_{M}^{R\gamma }$ and the similarity matrix $\Phi_{\Omega }^{R\gamma }$ are send to the Graph Neural Network (GNN) [28] $G_{a}$ for reasoning the appearance geometric features $X_{a}$ of the real samples, as shown in Figure 6c. Likewise, the normalized adjacency matrix ${\tilde{A}}_{a}$ of the $G_{a}$ is composed of $\Phi_{\Omega }^{R\gamma }$; $X_{a}^{l}$ and $X_{a}^{l+1}$ correspond to the input and output features of the $G_{a}$ respectively; $W_{a}^{l}$ is the learned weight matrix; and σ represents a ReLU activation function. The calculation formula is as follows:

(5) $$X_{a}^{l+1}=\sigma \left({\tilde{A}}_{a}X_{a}^{l}W_{a}^{l}\right)$$

### 2.4. Spatial Geometric Reasoning

**Figure 7:**
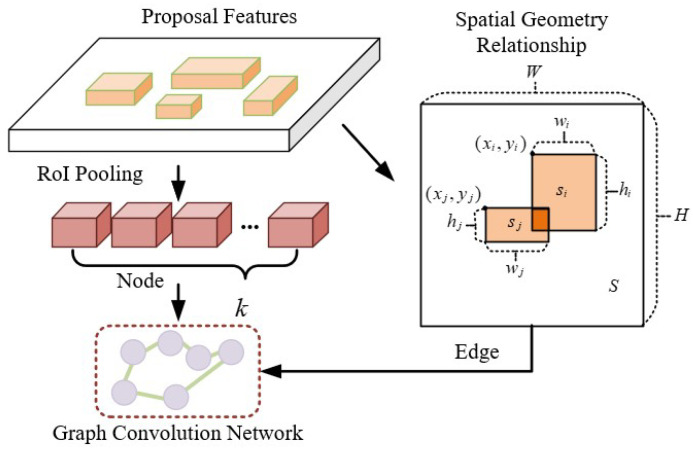
Architecture of the SGR model.

In aerial images of insulator defects, the insulator defect area is relatively small compared to the whole image, which makes defect detection more difficult. Therefore, to greater extract the defect features, it is necessary to design a module to capture the interaction information between the defect area and the global area. The obvious spatial information includes: (1) the defect area must be located on the main body of the insulator strings; (2) there is also a spatial positional relationship between different defects; (3) the spatial positional relationship also exists between the defect area and the adjacent insulators. By extracting the spatial information and utilizing the Graph Neural Network (GNN) for learning, the spatial geometry features for assisting the localization and regression of region proposals in the object can be obtained, as shown in Figure 7.

In detail, the proposal features were obtained from the real samples by RPN. Then it is submitted to RoI Pooling to generate the 128 regional proposals for a graph convolution network G:G=(ϑ,E). In the GNN, each node *v* corresponds to a region proposal. To reduce the redundant noise information in the modeling process of spatial geometric location information and make the output node information smooth, the *k* region proposals are selected as nodes θ of the GNN, where k is a hyperparameter; θ∈ϑ, ϑ is a set of nodes.

The spatial geometric relationship $r_{ij}$ between the region proposals is represented by a set of spatial location calculation laws. This enables the interaction of information between the region proposals and overcomes the effects of different scales of defects and different spatial locations. Due to the few defect labels in a single image, the spatial location relationship of the regional proposals with different labels is simple. Thus, only little node information is required to construct a graph neural network. The calculation formula is as follows:

(6) $$\begin{array}{c} r_{ij}=\left\{\frac{\left(x_{i}-x_{j}\right)}{w_{j}},\frac{\left(y_{i}-y_{j}\right)}{h_{j}},\frac{{\left(x_{i}-x_{j}\right)}^{2}}{w_{j}^{2}},\frac{{\left(y_{i}-y_{j}\right)}^{2}}{h_{j}^{2}},\right.\left.\log\left(\frac{w_{i}}{w_{j}}\right),\log\left(\frac{h_{i}}{h_{j}}\right)\right\} \end{array}$$

where $\left(x_{i},y_{i}\right)$ is a center point coordinates of the region proposals; $w_{i}$ and $h_{i}$ correspond to the width and height of the region proposals, respectively.

The spatial location relationship $r_{ij}$ is supplied to a ReLU activation function and then normalize it to obtain the final spatial geometry relationship $e_{ij}$. The spatial geometric relation $e_{ij}$ as the node-to-node edge in the GNN. The calculation formula is as follows:

(7) $$e_{ij}=\frac{exp(ReLU\left(W_{s}r_{ij}\right))}{\sum_{k}exp(ReLU\left(W_{s}r_{kj}\right))}$$

where $e_{ij}\in E\in R^{k\times k}$; *E* is a set of edges; $W_{s}$ is a learnable weight parameter.

After obtaining information about the nodes and edges used for the GNN, we construct a GNN $G_{s}$ to represent the spatial geometric features of the defect location information. where ${\tilde{A}}_{s}$ is the normalized adjacency matrix formed by $e_{ij}$; $X_{s}^{l}$ and $X_{s}^{l+1}$ correspond to the input features and output features of the GNN, respectively; $W_{s}^{l}$ is the learned weight matrix; σ represents the ReLU activation function. The calculation formula is as follows:

(8) $$X_{s}^{l+1}=\sigma \left({\tilde{A}}_{s}X_{s}^{l}W_{s}^{l}\right)$$

### 2.5. Training Method

This section describes the loss function, feature fusion approaches, and the model training process. The weighted sums operation is adopted to fuse the original features, the appearance of geometric features, and spatial geometric features. This can effectively avoid the impact of feature redundancy on the model performance. The calculation formula is as follows:

(9) $$f_{all}=f+\lambda X_{a}+\left(1-\lambda \right)X_{s}$$

where λ is a hyperparameter.

The loss function $L_{2}$ is a multi-task loss on each proposal RoI for jointly training classification and bounding-box regression. The calculation formula is as follows:

(10) $$L_{2}\left(p_{i},t_{i}\right)=\frac{1}{N_{cls}}\sum_{i}L_{cls}\left(p_{i},p_{i}^{*}\right)+\mu \frac{1}{N_{reg}}\sum_{i}p_{i}^{*}L_{reg}\left(t_{i},t_{i}^{*}\right)$$

where *i* is the index of proposals. The $p_{i}^{*}$ and $t_{i}^{*}$ are the ground-truth label and box, respectively. The classification loss $L_{cls}$ is cross entropy loss over multiple classes of fittings. The regression loss $L_{reg}$ is defined by $Smooth_{L_{1}}$ loss function. In practice, we adopt the generally used technique SGD to implement our algorithm.

The model training process is carried out with the following steps:

1. First, to obtain the deep features *f* of artificial defect samples, our method trains a classifier named Classifier-0 by cross-entropy loss $L_{1}$ in the preparatory phase;
2. Second, compute $\Sigma_{ij}^{\gamma }$ and $\Phi_{\Omega }^{\gamma }$ according to Equations (1) and (2), respectively.
3. Third, the Bboxs are obtained from the real samples through CNN, RPN, and RoI Pooling in turn by loss function $L_{2}$ in the formal phase;
4. Fourth, input the category information and feature information contained in Bboxs. Based on this information, $X_{a}$ is computed by Equations (4) and (5); $X_{s}$ is computed by Equations (6)–(8);
5. Fifth, fuse original features and enhanced features $X_{s}$ and $X_{a}$ together and output the $f_{all}$;
6. Last, train GNN $G_{s}$ and $G_{a}$ for reasoning and learning spatial geometric feature $X_{s}$ and appearance geometric feature $X_{a}$ by loss function $L_{2}$, respectively;.

## 3. Experiment and Numerical Result

### 3.1. Datasets Description

***AS-I Dataset:*** AS-I defect dataset is a high-quality database of different angles generated from 3D defect models, which was collected to solve the problem of insulator geometric defect detection. The dataset includes two categories of insulator defects, including 1175 damaged insulator images, 1814 missing insulator images, and 1360 defect-free insulator images. The resolution of each raw image is 1600×1200. All images are pure color backgrounds which makes the high contrast between object and background in the total dataset. Figure 4 shows a part of the AS-I dataset.

***RS-I Dataset:*** The RS-I defect dataset consists of 332 images of two types of defects, insulator damage and insulator missing, including 303 insulator labels, 165 missing insulator labels, and 183 damaged insulator labels. These defect images have different resolutions and most of them contain a series of noises, such as the diversity of defect shapes, and relatively low contrast between the object and the background. All these factors pose great challenges to detection. Figure 1 shows a part of the RS-I dataset.

### 3.2. Implementation Details

***Parameters Setting:*** We use Faster R-CNN as our baseline model and adopt a pre-trained ResNet50 [29] on ImageNet as the backbone network. In RS-I and AS-I datasets training stage, we both adopt mini-batch stochastic gradient descent (SGD) optimizer with a momentum of 0.9 for network optimization. In RS-I and AS-I datasets, the training set and the validation set are divided in the ratio of 1:1 (i.e., 166 training/test samples) Detailed parameters setting is shown in Table 1.

***Computation Platform:*** We implement our method on the PyCharm with the open-source toolbox PyTorch. We run our method in an NVIDIA GTX 1080Ti GPU on Ubuntu 16.04.

### 3.3. Evaluation Metrics

In terms of evaluation metrics, binary precision (*P*) and recall (*R*) are chosen to validate the detection performance in our work. The binary *P* and *R* are calculated by:

(11) $$P=\frac{TP}{TP+FP}$$

(12) $$R=\frac{TP}{TP+FN}$$

where *TP*, *FP* and *FN* represent the number of true positive, false positive and false negative samples respectively.

Furthermore, average precision (*AP*) for binary pest localization is applied as a comprehensive evaluation metric to take the *P* and *R* into consideration together. In single object detection, the AP of detecting an object is computed by the integration of the precision-recall (*PR*) curve. The calculation formula is as follows:

(13) $$AP=\int_{0}^{1}PdR$$

In the multi-object detecting task, we usually select the mean average precision (*mAP*) that is obtained by taking an average of *APs* from all the fitting categories to evaluate the model accuracy. The calculation formula is as follows:

(14) $$mAP=\frac{1}{C}\sum_{i=1}^{C}AP_{i}$$

For a comprehensive and comprehensive evaluation, we adopt the metrics from COCO detection evaluation criteria [30], i.e., mAP across different intersection over union (IoU) thresholds (IoU = 0.5:0.95, 0.5, 0.75). We also use average recall (AR) with a different number of given detections per image (1, 10).

### 3.4. Comprehensive Comparison and Analysis

For the sake of verifying the performance of the proposed method more comprehensively, The COCO evaluation criteria are employed to compare MGRN with other object detection models, viz. YOLOv3 [22], Retina Net [31], SSD [21], Cascade R-CNN [32], Libra R-CNN [33] and baseline model. The results are listed in Table 2.

**Figure 8:**
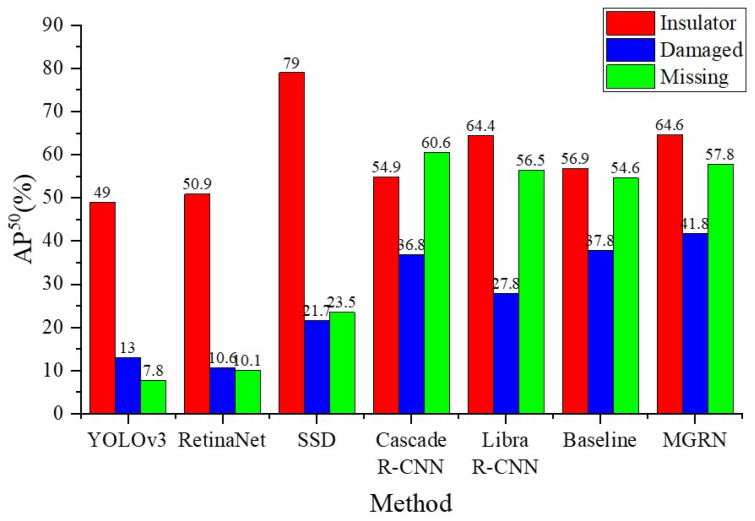
Comparison of defect detection results of various models.

It can be seen from Table 2 that the $AP^{50}$ value of the MGRN model reached 54.7%, an increment of 4.9% compared with the baseline model. Compared with Libra R-CNN and Cascade R-CNN model, the $AP^{50}$ value of our model was increased by 3.9% and 5.1%, respectively. This demonstrates that our method can improve both the accuracy and false discovery rate by extracting the appearance geometric features and spatial geometric features of defects. We also compare our method to other object detection models in Table 2. It can be observed that the MGRN outperforms other competing methods by a large margin. Compared with other models, MGRN has greatly improved the detection effect of hard-detection insulator defects. In terms of damaged defect detection, the proposed MGRN increases the $AP^{50}$ by 4% compared with the baseline model. Similarly, the proposed MGRN increases the $AP^{50}$ by 3.2% compared with the baseline model in the missing defect detection. The detection results of each category are shown in Figure 8.

**Figure 9:**
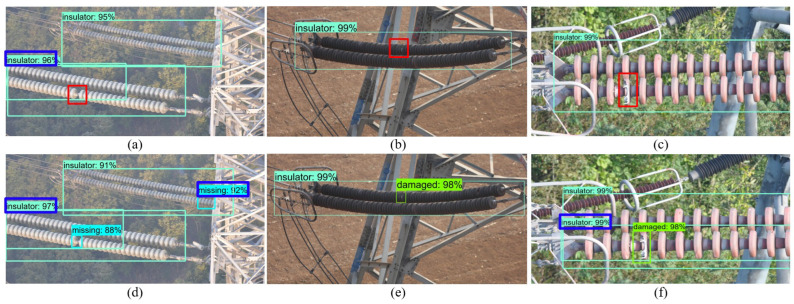
Qualitative result comparison for defects detection. (**a**–**c**) baseline detection results; (**d**–**f**) MGRN detection results.

To qualitatively analyze the detection effect of the proposed model, the visualization comparison between the baseline and MGRN is shown in Figure 9, where the blue boxes represent the repeated detection of insulator and insulator defects and the red boxes represent the missed-detected insulator defects. As shown in Figure 9, damaged and missing defects can be hardly detected due to the size of the defect in the image being too small.

In particular, the similarity in shape and background of defects and adjacent insulators results in missed detections in the baseline model. MGRN effectively utilizes the appearance geometric information and spatial geometric information of defects, reduces the influence of the texture color information on the model, and greatly improves the detection precision. As shown in the red box in Figure 9b, defects with minor features and similar background colors are not detected by the baseline model. However, the MGRN model overcomes this problem in Figure 9e, and it is able to detect this defect.

### 3.5. Ablative Study

To evaluate the proposed solution, this article conducts a rank of ablative experiments, including hyperparameter setting in the total model, and the effects of the fusion feature. We did some tests on the Faster R-CNN baseline model. All the evaluations of these ablative experiments are based on the RS-I dataset.

***Contributions of Each Sub-module:*** The proposed MGRN consists of three sub-modules, i.e., AGR sub-module, PFT sub-module, and SGR sub-module. The AGR sub-module and PFT sub-module are used to extract the appearance geometric features of defects. The SGR module is used to extract the spatial geometric features of defects. We compare the detection results of different sub-modules on the RS-I dataset. The result is shown in Table 3. The AGF represents the model of only using the AGR sub-module and the PFT sub-module for defect detection. The SGF represents the model of only using the SGR sub-module for defect detection. It can be observed that the performance will increase by 3.2% and 4.2% if we add either sub-network, which can further validate the effectiveness of each component in the proposed MGRN.

***Impact of Different Regional Proposal Parameters:*** we compare the detection performance with the different number of region proposals based on the SGR sub-module. The hyperparameter $k\in \left\{2,4,8,16,32,64,128\right\}$. Table 4 shows the $AP^{50}$ result with different *k*, marking best and second best. It can be observed in Table 4 that the SGR sub-module can preferably improve the baseline model when only the four region proposals are used to represent the spatial geometric features. At this point, the $AP^{50}$ value of the MGRN model with only the SGR sub-module reached 53.1%. It can be observed that as the value of *k* increases, the performance of the model declines instead. When all 128 region proposals are used to structure the network, the SGR sub-module accomplishes the maximum results in $AP^{50}$, $AP^{75}$, $AR^{1}$, and $AR^{10}$. However, this will generate redundant information and a huge computational overhead. Thus, we choose the k=4 in the SGR sub-module.

**Figure 10:**
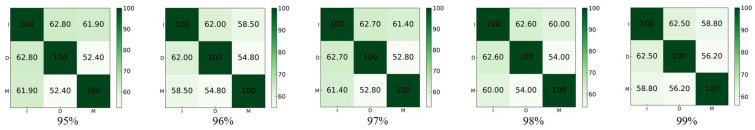
The similarity matrix with different accuracy.

***Impact of Different Similarity Matrix with Different Accuracy Levels:*** We use different accuracy to conduct the ablative experiments from 95% to 99%, the accuracy $\gamma \in \left\{95\%,96\%,97\%,98\%,99\%\right\}$ Classifiers with different accuracy determine the results of appearance geometric feature extraction for artificial samples. We calculated the similarity matrix generated in the AGC sub-module with different precision, as shown in Figure 10. It can be observed in Figure 10 that $S_{I-D}$ is greater than $S_{I-M}$ and is greater than $S_{D-M}$ at different accuracy.

To prove the effectiveness of appearance geometric feature generated by AGC sub-module and PFT sub-module with different accuracy of the similarity matrix. The results are shown in Table 5. The $AP^{50}$ value of the model gradually increases as the accuracy γ improves. When the accuracy γ is 99%, the $AP^{50}$ value of the MGRN model with only the AGR sub-module and PFT sub-module reached 54%. This shows that the appearance of geometric features from the artificial samples can be effectively expressed through the graph reasoning network $G_{a}$, thereby improving the insulator defects detection performance.

**Figure 11:**
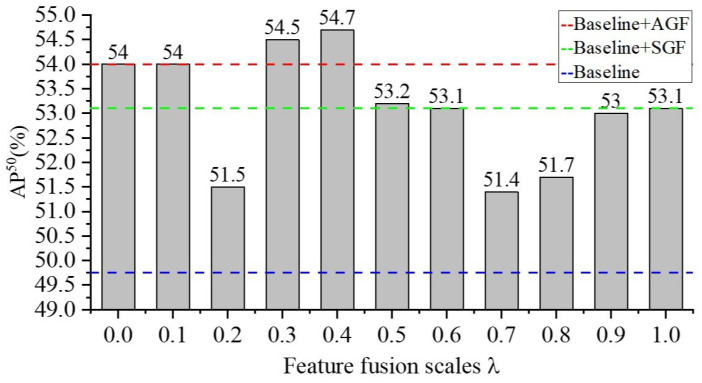
Performance of different feature fusion scales.

***Impact of Different Features Fusion Scales:*** This work evaluates the effect of different feature fusion scales on the MGRN model, and the corresponding performance is shown in Figure 11. It can be observed from Figure 11 that the MGRN model displays better and reaches 54.7% when the hyperparameter λ equals 0.4, which demonstrates the feature maps fusion can significantly promotes the performance of the detection in the proposed solution. Figure 11 shows that the AGR sub-module, PFT sub-module, and SGR sub-module can effectively solve the problem of insulator geometric defect detection with strong robustness. In addition, the large gap in feature fusion rate will reduce the detection performance of the MGRN model as shown in Figure 11. However, the overall performance of the model still improves over the baseline model.

## 4. Conclusive Remarks

This paper developed an automatic reasoning detection network based on multi-geometric features for defect detection of electric transmission line insulators. In the proposed solution, the AGR sub-module is developed to extract the appearance geometric features of defects from the artificial insulator samples with defects. Through designing the PFT sub-module, the extracted appearance geometric features are made available to the graph convolutional network for reasoning learning. In addition, the SGR sub-module is developed to identify the space geometric position relationship between defects to capture the interaction information under the regional proposals.

The proposed solution is extensively assessed through experiments against the existing solutions and the numerical results demonstrated that the proposed MGRN-based solution significantly advanced the benchmarking solutions on insulator geometric defect detection with limited data availability, and the $AP^{50}$ improvement of the scarcity sample is up to 41.8%. To our knowledge, it is the first work that artificial samples are expressed in deep space and then transformed into real samples and applied to transmission line insulator geometric defect detection. In future research, we plan to seek a better appearance geometric representation method of defects to improve the detection performance and meet the requirements of real-world scenarios.

## Figures and Tables

**Table 1:** Experimental basic parameters setting.

Parameters Setting	RS-I Dataset	AS-I Dataset
Backbone	Resnet50	Resnet34
Optimizer	SGD	SGD
Batch size	1	16
Epoch	50	100

**Table 2:** Performance comparison of different detection methods. Bolded numbers indicate optimal.

Method	${\mathrm{AP}}^{50:95}$	${\mathrm{AP}}^{50}$	${\mathrm{AP}}^{75}$	${\mathrm{AR}}^{1}$	${\mathrm{AR}}^{10}$
YOLOv3 [22]	8.4	23.3	5.2	15.7	24.8
Retina Net [31]	10.3	23.9	7.0	16.9	26.3
SSD [21]	18.0	41.4	13.8	23.1	30.2
Cascade R-CNN [32]	24.1	49.6	18.2	29.0	36.0
Libra R-CNN [33]	20.1	50.8	11.2	26.9	36.6
Baseline [24]	23.9	49.8	18.0	30.6	34.7
MGRN (ours)	**25.4**	**54.7**	**24.6**	**31.8**	**37.8**

**Table 3:** Comparison of test results of different sub-modules. Bolded numbers indicate optimal.

Method	${\mathrm{AP}}^{50}$	${\mathrm{AP}}^{75}$	${\mathrm{AR}}^{1}$	${\mathrm{AR}}^{10}$
Baseline	49.8	18.0	30.6	34.7
Baseline+SGF	53.1	22.9	30.3	36.7
Baseline+AGF	54.0	21.1	**32.5**	36.7
MGRN (ours)	**54.7**	**24.6**	31.8	**37.8**

**Table 4:** Comparison of test results of different *k*. The underlined numbers indicate sub-optimal, the bolded numbers indicate optimal.

*k*	${\mathrm{AP}}^{50}$	${\mathrm{AP}}^{75}$	${\mathrm{AR}}^{1}$	${\mathrm{AR}}^{10}$
2	51.8	22.4	30.9	35.6
4	**53.1**	22.9	30.3	36.7
8	50.0	18.5	30.3	34.0
16	50.2	18.7	28.6	33.5
32	50.6	18.7	29.4	34.3
64	51.7	14.9	30.3	36.0
128	51.5	**25.8**	**32.7**	**37.7**

**Table 5:** Comparison of test results of different accuracy.

Accuracy γ	${\mathrm{AP}}^{50}$	${\mathrm{AP}}^{75}$	${\mathrm{AR}}^{1}$	${\mathrm{AR}}^{10}$
95%	50.0	19.3	32.5	37.4
96%	51.1	18.0	31.4	37.6
97%	52.4	21.4	31.9	37.2
98%	52.9	22.2	32.1	36.0
99%	54.0	21.1	32.5	36.7

## Data Availability

Not applicable.

## Abbreviations

The following abbreviations are used in this manuscript:

MGRN	Multi-Geometric Reasoning Network
SGR	Spatial Geometric Reasoning
AGR	Appearance Geometric Reasoning
PFT	Parallel Feature Transformation
SSD	Single Shot MultiBox Detector
CNN	Convolutional Neural Network
YOLO	You Only Look Once
RPN	Regional Proposal Network
GPU	Graphics Processing Unit
P	Precision
R	Recall
TP	True Positive
FP	False Positive
FN	False Negative
UAVs	Unmanned Aerial Vehicles
